# A 6‐year prospective clinical cohort study on the bidirectional association between frailty and depressive disorder

**DOI:** 10.1002/gps.5588

**Published:** 2021-06-19

**Authors:** Richard C. Oude Voshaar, Menelaos Dimitriadis, Rob H. S. vandenBrink, Ivan Aprahamian, Marcus K. Borges, Radboud M. Marijnissen, Emiel O. Hoogendijk, Didi Rhebergen, Hans W. Jeuring

**Affiliations:** ^1^ Department of Psychiatry University of Groningen University Medical Center Groningen Groningen The Netherlands; ^2^ Department of Internal Medicine Geriatrics Division Faculty of Medicine of Jundiaí Jundiaí Brazil; ^3^ Department and Institute of Psychiatry São Paulo University of São Paulo Brazil; ^4^ Department of Epidemiology and Biostatistics Amsterdam UMC – Location VU University Medical Center Amsterdam The Netherlands; ^5^ Department of Psychiatry Netherlands & GGZ Ingeest Specialized Mental Health Care Amsterdam UMC – Location VU University Medical Center Amsterdam The Netherlands

**Keywords:** ageing, biological ageing, depressive disorder, frailty, frailty index

## Abstract

**Introduction:**

Depressive disorder has been conceptualised as a condition of accelerated biological ageing. We operationalised a frailty index (FI) as marker for biological ageing aimed to explore the bidirectional, longitudinal association between frailty and either depressive symptoms or depressive disorder.

**Methods:**

A cohort study with 6‐year follow‐up including 377 older (≥60 years) outpatients with a DSM‐IV‐defined depressive disorder and 132 never‐depressed controls. Site visits at baseline, 2 and 6‐year follow‐up were conducted and included the CIDI 2.0 to assess depressive disorder and relevant covariates. Depressive symptom severity and mortality were assessed every 6 months by mail and telephone. A 41‐item FI was operationalised and validated against the 6‐year morality rate by Cox regression (HR_FI_ = 1.04 [95% CI: 1.02–1.06]).

**Results:**

Cox regression showed that a higher FI was associated with a lower chance of remission among depressed patients (HR_FI_ = 0.98 [95% CI: 0.97–0.99]). Nonetheless, this latter effect disappeared after adjustment for baseline depressive symptom severity. Linear mixed models showed that the FI increased over time in the whole sample (*B*[SE] = 0.94 (0.12), *p* < .001) with a differential impact of depressive symptom severity and depressive disorder. Higher baseline depressive symptom severity was associated with an attenuated and depressive disorder with an accelerated increase of the FI over time.

**Conclusions:**

The sum score of depression rating scales is likely confounded by frailty. Depressive disorder, according to DSM‐IV criteria, is associated with accelerated biological ageing. This argues for the development of multidisciplinary geriatric care models incorporating frailty to improve the overall outcome of late‐life depression.

## Key Points

- To disentangle the association between frailty and depression, depression should be diagnosed according to diagnostic criteria, as scores on depression rating scales are confounded by frailty
- Depressive disorder is associated with accelerated ageing, as indexed by the frailty index

## 1 INTRODUCTION

Frailty and late‐life depression are common and often co‐occurring conditions in later life. Frailty is a state of vulnerability characterised by poor resolution of homeostasis following a stressor, which places persons at risk of iatrogenic harm, dependency and death.^1^ About one in 10 adults aged 65+ years suffer from frailty, with a sharp increase after age 75.^2^ Frailty has been related to depression in later life. Among persons aged 75+ years, the pooled prevalence rate of depressive disorder is 7.2% and of clinically relevant depressive symptoms 17.1%.^3^ Meta‐analysis has shown that 40.4% of depressed older persons are frail and that 38.6% of frail persons are depressed, with sparse longitudinal data suggesting a bidirectional association between both conditions.^4^ The strength of this association might be explained partly by confounding, because in most studies, depression has been assessed only with self‐report questionnaires and the criteria of frailty and depression partly overlap.^4^ In clinical practice, misclassifying frailty for depression or vice versa places patients at risk of inappropriate treatment, since both diagnoses refer to different treatment guidelines and are generally treated in different healthcare settings.^1^

A common model of frailty is the deficit accumulation model.^5^, ^6^, ^7^ This model postulates that the proportion of at least 30 ageing‐related health deficits, that is, the frailty index (FI), reflects biological age on top of chronological age.^6^, ^7^ The conceptualisation of the FI as a clinical marker of biological ageing is interesting. Depression has also been conceptualised as a disorder of accelerated ageing, based on its association with several biomarkers of cellular ageing, the onset of chronic somatic diseases and premature mortality.^8^ In population‐based cohort studies, factor analyses have shown that frailty and depression represent different, but strongly associated dimensions, 9 whereas latent class analyses have shown that criteria for frailty and depression generally identify the same subgroup of persons.^10^ These data suggest that frailty and depression are two sides of the same coin, which may have contributed to the neglect of frailty in geriatric mental health care. The few studies on frailty in mental healthcare populations, however, show widely different prevalence rates, that is, 27% among depressed outpatients^11^ and between 53% and 84% among psychiatric inpatients.^12^, ^13^ Collectively, these data suggest that it might be important to formally diagnose depressive disorder according to prevailing classification systems using diagnostic interviews instead of diagnosing depression based on self‐report questionnaires.

For the present study, we operationalised a FI within the Netherlands Study of Depression in Older persons (NESDO) cohort in order to (1) study the prevalence of frailty among depressed patients, (2) study the impact of frailty on the outcome of depression and (3) to study the impact of depression on the course of frailty. We hypothesize that (a) the FI predicts a protracted course of depression^14^ and that (b) the FI increases significantly more over time among patients suffering from depressive disorder compared to a never depressed comparison group.

## 2 MATERIALS AND METHODS

### 2.1 Study design and sample

The study was embedded in the NESDO cohort. For a full description of this prospective cohort study, as well as the references for all of the measurements used, we refer to Comijs et al. and Jeuring et al.^15^, ^16^ NESDO included 378 depressed subjects with one or more depressive disorder(s), and a comparison group of 132 never‐depressed older persons. Depressive disorders were diagnosed according to DSM‐IV‐TR criteria using the Composite International Diagnostic Interview (CIDI version 2.1). Persons with a (suspected or established) diagnosis of dementia, a Mini Mental State Examination (MMSE) score under 18, a psychotic disorder or insufficient mastery of the Dutch language were excluded.^15^

Trained research assistants conducted site visits at baseline, 2‐year and 6‐year follow‐up. Data were gathered about demographic characteristics, mental health outcomes, prescribed drug use, and psychosocial, biological, cognitive and genetic determinants using structured interviews, cognitive tests and a physical examination including blood collection.^15^, ^16^ Every 6 months, questionnaires were sent by mail to the participants to assess depressive symptom severity.

The authors assert that all procedures contributing to this work comply with the ethical standards of the relevant national and institutional committees on human experimentation and with the Helsinki Declaration of 1975, as revised in 2008. The protocol of the NESDO study was approved centrally by the Ethical Review Board of the VU University Medical Center. All participants provided written informed consent after receiving detailed oral and written information.^15^

### 2.2 Depression

#### 2.2.1 Depressive disorder

At baseline, 2‐ and 6‐year follow‐up, the CIDI version 2.1 was used to diagnose the presence of a major depressive disorder and dysthymia in the previous 6 months according to DSM‐IV‐TR criteria. Additional questions were added to diagnose past‐month minor depression according to the research criteria of the DSM‐IV‐TR.^15^

#### 2.2.2 Depressive symptom severity

The severity of depressive symptoms was assessed every 6 months by means of the 30‐item, self‐report Inventory of Depressive Symptoms (IDS).

### 2.3 Frailty

Since the NESDO study has sufficient data on health deficits, we were able to operationalise an FI following the guidelines described by Searle and colleagues.^6^ The FI is the ratio of health deficits present to the total number of deficits considered. Deficits in health can be symptoms, signs, diseases and disabilities, as well as laboratory, radiographic or electrocardiographic abnormalities.^6^ Irrespective of the specific health deficits and the number of health deficits taken into account, the FI is a better predictor of adverse health outcomes than chronological age and other indices of biological age.^5^, ^17^ In general, patients scoring 0.25 or above are considered to be frail. Because of its continuous nature, the FI is sensitive to change and makes it possible to study trajectories of frailty over time.^18^, ^19^

The NESDO‐FI was operationalised according to the guidelines by Searle et al.^6^ All variables, known to be associated with age and measured at baseline 2‐ and 6‐year follow‐up, were considered of interest. These variables covered (1) chronic somatic diseases, (2) objective measures of physical performance, (3) subjective measures of physical and cognitive performance, (4) blood‐born biomarkers, (5) sensory functioning, (6) subjective health measures and, finally, (7) cognitive functioning. We a priori excluded mood to prevent interference with depressive disorder. Furthermore, some depression‐related deficits (anxiety, apathy and sleep) were also excluded since the assumptions for including were not met. As shown in Appendix SA, 41 out of 48 potentially relevant health deficits satisfied the criteria for inclusion in an FI as specified by Searle et al.^6^ Validity of the NESDO‐FI was supported by its associations with chronological age, sex and mortality as the gold standard (see Table 1). The NESDO‐FI had a normal distribution, had a maximum value of 0.56 in our population and correlated significantly with chronological age. These characteristics neither differed meaningfully between baseline and follow‐up assessments nor between depressed patients and non‐depressed persons, except that the association between the NESDO‐FI and chronological age at baseline was lower in the depressed subgroup (*r* = 0.28, *p* < .001) compared to the non‐depressed comparison group (*r* = 0.52, *p* < .001). Furthermore, the FI at baseline did not differ by sex (unadjusted analyses: *t* = 0.2, df = 507, *p* = 0.847; adjusted for the covariates including the presence of a depressive disorder: *F* = 1.5, df = 1, *p* = .221). In NESDO, mortality was evaluated every 6 months among patients who dropped out.

As shown in Table 1, Cox‐regression analysis showed that a higher FI as well as a higher chronological age was independent predictors of mortality over a 6‐year follow‐up. The interaction of the FI with depression status at baseline was not significant (*B*[SE] = −0.058 (0.046), *p* = 0.213), which indicates that this effect of frailty did not differ between depressed patients and their non‐depressed counterparts.

**TABLE 1:** Association of the NESDO Frailty Index as marker for biological age as well as chronological age with the 6‐year mortality rate by Cox regression

Statistical models	HR [95% CI]	*p*‐Value
Unadjusted models		
*Model 1*		
Frailty index^a^	1.05 [1.03–1.07]	<.001
*Model 2*		
Age (years)	1.07 [1.04–1.11]	<.001
*Model 3*		
Frailty index^a^	1.04 [1.02–1.06]	.001
Age (years)	1.05 [1.02–1.09]	.001
Fully adjusted model^b^		
Frailty index^a^	1.04 [1.00–1.08]	.037
Age (years)	1.06 [1.02–1.10]	.002

Abbreviations: CI, confidence interval; HR, hazard ratio.

^a^ The HR per 0.01 on the frailty index.

^b^ Adjusted for gender, education, alcohol, smoking, physical activity, BMI, chronic diseases, cognition and depression status.

### 2.4 Covariates

Covariates were chosen based on their association with either mortality or late‐life depression. In all of the fully adjusted models described below, we included socio‐demographic characteristics (age, sex and years of education), lifestyle characteristics (average number of alcoholic drinks per day, currently smoking, physical activity in MET‐minutes as measured with the International Physical Activity Questionnaire short form [IPAQ‐SF] and waist circumference in centimeters), global cognitive functioning (as measured with the MMSE), and the total number of chronic somatic diseases (i.e., lung disease, cardiac diseases, liver disease, atherosclerotic disease, cerebrovascular disease, hypertension, diabetes mellitus, thyroid disease, malignant neoplasms and osteoarthritis). In addition, the analyses of the course of depression were adjusted for the use of antidepressants.

### 2.5 Statistical analyses

The characteristics of the FI was explored by a histogram and by calculating the mean FI score and standard deviations, skewness and kurtosis, range, and Pearson's correlation with chronological age for the total study population, as well as stratified by depression status. Sex differences were explored by *t*‐test statistics and ANCOVA adjusted for covariates.

Bivariate and multivariate Cox proportional hazard models were subsequently fitted to study the association between the FI (multiplied by 100 for interpretation) and all‐cause mortality (censored at six 6‐year follow‐up or earlier in case dropout for another reason than death).

Within the depressed subgroup, the FI was examined as a predictor for remission at 2‐ and 6‐year follow‐up using Cox regression. The bivariate model as well as multivariate models with and without additional correction for baseline depressive symptom severity were conducted.

To test whether the FI is associated with the course of depressive symptoms, we performed random coefficient mixed‐effect models with the IDS sum score (every 6 months) as the dependent variable, and the FI at baseline as independent variable among the depressed subgroup. To determine the best‐fitting random coefficient model, the likelihood ratio test was used to compare models with random coefficients for intercept and/or slope per subject. Presence of an association was tested by the interaction of the independent variable (FI) with the variable ‘time’, which indicated the assessment of depressive symptoms at 6‐month intervals. We included both time and time^2^ in order to explore linear as well as non‐linear effects.

Next, in the entire study population, we studied whether depression was associated with the course of frailty over time using random coefficient mixed‐effect models (as described above), but now with the FI at baseline, 2‐year or 6‐year follow‐up as the dependent variable. We tested both, a continuous model with the severity of depressive symptoms at baseline (IDS sum score) as the independent variable as well as a categorical disease model with the presence of a depressive disorder (yes/no) at baseline as the independent variable.

All analyses were conducted in SPSS version 24. *p*‐Values of less than .05 were considered statistically significant.

## 3 RESULTS

### 3.1 Sample

The FI was available at baseline for 509 participants, at 2‐year follow‐up for 387 participants, and finally at 6‐year follow‐up for 279 participants, resulting in 1175 observations. Participants who dropped out at the 2‐ and 6‐year follow‐ups were significantly more frail at baseline (2‐year follow‐up: 0.21 [SD = 0.10] versus 0.27 [SD = 0.11], *t* = −5.5, df = 507, *p* < .001; 6‐year follow‐up: 0.19 [SD = 0.09] versus 0.27 [SD = 0.11], *t* = −8.1, df = 507, *p* < .001). Table 2 presents all baseline characteristics, stratified by depression status.

**TABLE 2:** Baseline characteristics, stratified by the presence of a DSM‐IV‐TR defined depressive disorder

	Sample			
	Overall (*n* = 509)	Non‐depressed (*n* = 132)	Depressed (*n* = 377)	Statistics
Socio‐demographics				
Mean (SD) age (years)	70.5 (7.3)	70.1 (7.2)	70.7 (7.4)	*t* = −0.9, df = 507, *p* = .388
Female, *n* (%)	330 (64.8)	81 (61.4)	249 (66.0)	Chi^2^ = 0.9, df = 2, *p* = .332
Mean (SD) years of education	11.0 (3.6)	12.5 (3.5)	10.4 (3.4)	*t* = 5.8, df = 507, *p* < .001
Lifestyle characteristics				
Mean (SD) alcohol units/day	0.7 (1.0)	1.1 (1.1)	0.6 (0.9)	*t* = 5.1, df = 497, *p* < .001
Currently smoking (yes), n (%)	111 (21.9)	11 (8.3)	100 (26.7)	Chi^2^ = 19.2, df = 2, *p* < .001
Mean (SD) physical activity (MET‐min)	2636 (2614)	3324 (2909)	2395 (2462)	*t* = 3.5, df = 492, *p* = .001
Mean (SD) waist circumference (cm)	94.6 (13.1)	97.6 (12.7)	93.5 (13.0)	*t* = 3.1, df = 503, *p* = .002
Cognitive and physical characteristics				
Mean (SD) cognitive functioning (MMSE)	27.9 (1.9)	28.3 (1.6)	27.7 (2.0)	*t* = 3.4, df = 507, *p* = .001
Mean (SD) number somatic diseases	2.4 (1.6)	1.9 (1.3)	2.5 (1.7)	*t* = −3.9, df = 507, *p* < .001
Mean (SD) frailty index	0.23 (0.10)	0.17 (0.08)	0.25 (0.11)	*t* = −7.3, df = 507, *p* < .001
Frailty (FI ≥ 0.25), n (%)	191 (37.5)	17 (12.9)	174 (46.2)	Chi^2^ = 46.2, df = 1, *p* < .001

Abbreviations: FI, frailty index; MMSE, Mini Mental State Examination; SD, standard deviation.

Depressed patients were significantly more frail compared to non‐depressed persons at baseline (0.25 [SD = 0.11] versus 0.17 [SD = 0.08], *t* = −7.3, df = 507, *p* < .001). This difference remained significant when adjusted for covariates (*F* = 29.7, df = 1, *p* < .001). Based on a cut‐off of 0.25, a total of 174/377 (46.2%) depressed patients and 17/132 (12.9%) non‐depressed participants were frail (Chi^2^ = 46.2, df = 1, *p* < .001).

### 3.2 Impact of frailty on course of depression

Within the depressed subgroup, Cox ‐regression showed that a higher FI lowered the chance of remission in the unadjusted model (HR_FI_ = 0.98 [95% CI: 0.97–0.99], *p* = .003). This effect became non‐significant in the fully adjusted model, in which only baseline severity of depressive symptoms remained statistically significant (HR_IDS_ = 0.98 [95% CI: 0.96–0.99], *p* < .001).

Furthermore, linear mixed models within the depressed subgroup showed that the FI at baseline was not associated with the course of depressive symptoms over time. The time by frailty interaction was not significant in the unadjusted (*B*[SE] = −0.74 [SE = 0.58], *p* = .199) or the adjusted model (*B*[SE] = −0.64[SD = 0.59], *p* = .281).

### 3.3 Impact of depression on course of frailty

Linear mixed models showed a significant time effect (unadjusted *B*[SE] = 0.899[0.121], *p* < .001; adjusted: *B*[SE] = 0.939[0.122], *p* < .001), indicating that the FI increases over time. The interaction of time by depressive symptom severity was also significant (unadjusted: *B*[SE] = −0.013[0.005], *p* = .004; adjusted: *B*[SE] = −0.013[0.005], *p* = .004), indicating that higher baseline depressive symptom severity was associated with a lower increase of the FI over time. As shown in Table 3, we also found an additional quadratic time effect, but this effect was independent of baseline depressive symptom severity.

**TABLE 3:** Linear mixed models of the predictive value of depression on the course of the frailty index over a 6‐year follow‐up

Continuous depression model	Unadjusted model 1			Unadjusted model 2			Fully adjusted model^a^		
	*F*	df	*p*	*F*	df	*p*	*F*	df	*p*
IDS (depressive symptoms)	175.4	478.8	<.001	175.3	500.9	<.001	107.3	481.2	<.001
Time	17.9	389.2	<.001	33.9	509.9	<.001	29.0	528.6	<.001
Time^2^	4.7	386.6	.030	7.4	362.9	.007	4.0	443.1	.046
IDS by time	3.5	390.8	.062	9.7	324.3	.002	7.1	256.2	.008
IDS by time^2^	1.3	394.3	.258	‐	‐	‐	‐	‐	‐

Categorical disease model	Unadjusted model			IDS‐adjusted model^b^			Fully adjusted model^c^		
	*F*	df	*p*	*F*	df	*p*	*F*	df	*p*
Depressive disorder	50.6	615.7	<.001	6.9	777.0	.009	3.7	856.8	.056
Time	13.5	670.3	<.001	28.4	654.4	<.001	24.7	695.0	<.001
Time^2^	2.3	665.2	.131	7.4	644.8	.007	5.3	681.5	.022
Depressive disorder by time	0.2	670.3	.232	4.1	657.3	.042	4.7	698.6	.030
Depressive disorder by time^2^	0.6	665.2	.625	3.3	645.9	.071	4.6	682.7	.033

^a^ Adjusted for age, gender, education, smoking, alcohol use, physical activity, waist circumference, cognitive functioning and somatic diseases.

^b^ The IDS (severity of depressive symptoms) is added as a time‐varying covariate.

^c^ The categorical model is adjusted for the same covariates and additionally adjusted for the severity of depressive symptoms as a time‐varying covariate.

**FIGURE 1:**
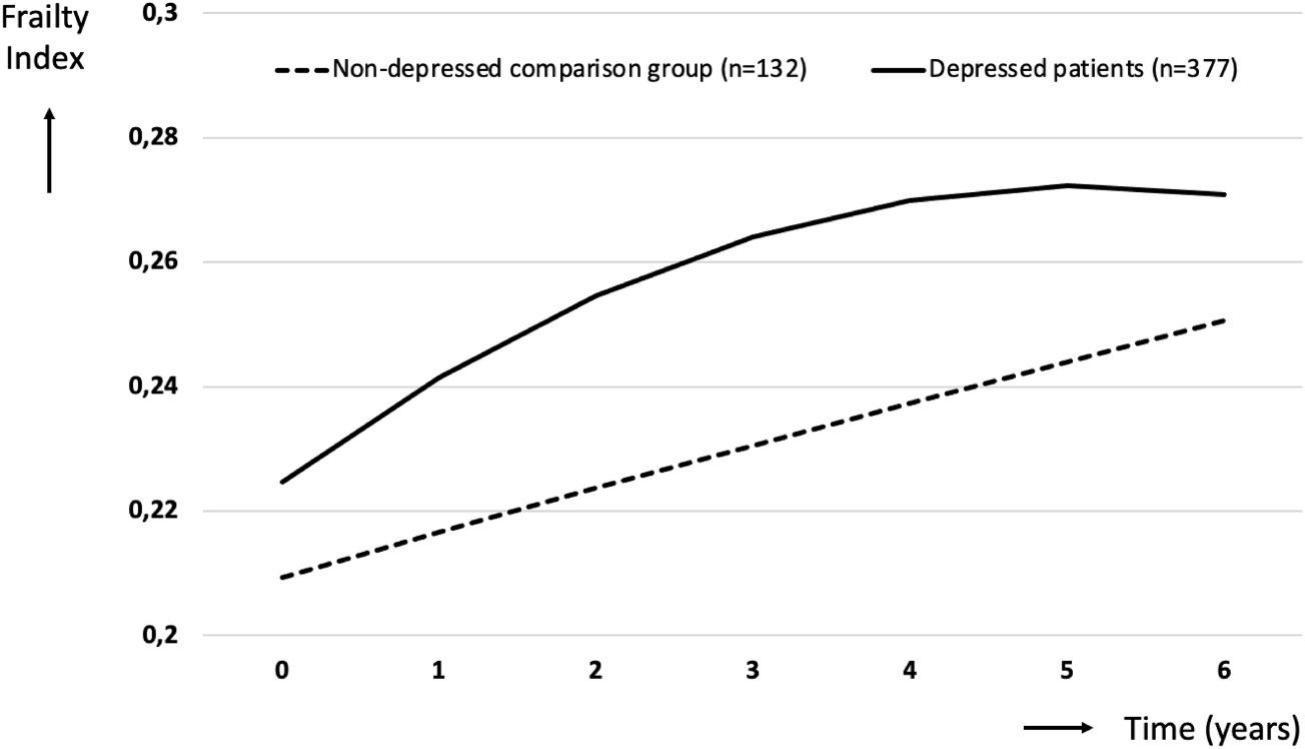
Estimated course of the frailty index over time (per year, adjusted for covariates), stratified by the presence of a depressive disorder

The analyses on the impact of baseline depressive symptom severity on the course of frailty suggest that the FI is confounded by depressive symptom severity. We therefore included depressive symptom severity as a time‐dependent covariate when studying the impact of depressive disorder on the course of frailty. The fully adjusted linear mixed models showed a significant interaction between the presence of a depressive disorder with both time and time^2^ (see Table 3). Figure 1 presents the estimated course of the FI for persons with and without a depressive disorder adjusted for confounders.

## 4 DISCUSSION

### 4.1 Main findings

Depressed patients were more often frail and had substantially higher frailty scores compared to their non‐depressed counterparts. A higher frailty severity was also associated with non‐remission at follow‐up within the depressed subgroup, but this effect was fully explained by a higher baseline depressive symptom severity. Interestingly, a depressive disorder according to DSM‐IV criteria predicted an accelerated increase of frailty over time, adding further evidence to the hypothesis of depression as a disorder of accelerated ageing.

### 4.2 Validity of the NESDO‐FI

Within a large sample of clinically depressed older patients and a never‐depressed comparison group, we operationalized an FI (NESDO‐FI) that behaves similarly to FI operationalised in population‐based cohort studies, that is, a positive association with chronological age and female sex. The NESDO‐FI had a normal distribution, which contrasts with the distribution of the FI in population‐based cohorts, but which is consistent with previous papers on the FI in acute ill and hospital‐based populations.^6^, ^7^

Furthermore, the NESDO‐FI was associated with mortality independent of chronological age. The hazard ratio (HR) for mortality of the NESDO‐FI was 1.04, like the pooled HR of 1.039 (95% CI: 1.033–1044) found in a recent meta‐analysis.^20^

The predictive validity of the NESDO‐FI is clinically important because depressive disorder itself is also considered a risk factor for premature mortality. Contrary to a causative effect, the most recent meta‐analysis showed that the HR decreased in magnitude and lost statistical significance when restricted to well‐controlled, high‐quality, community‐based studies on DSM‐defined depressive disorders.^21^ To our knowledge, only one study on excess mortality due to depressive disorder has adjusted for frailty. That study reported a crude mortality hazard of 4.3 for depression among males aged 75 and over, dropping to 1.8 after additional correction for a self‐report frailty questionnaire.^22^

### 4.3 Increase of frailty over time

In line with the limited number of prospective studies on the FI, the NESDO‐FI increased significantly over time.^19^, ^23^, ^24^, ^25^ The increase in frailty of 1% per year that we found is slightly lower than the assumed average increase of health deficits of 3% per year found in cross‐sectional studies comparing age groups^26^ as well as in some longitudinal studies,^23^, ^24^ but is comparable to the values found in longitudinal cohort studies across Europe.^19^, ^25^ However, we did not find a linear or exponential increase of frailty over time. Our non‐linear quadratic effect was driven by the depressed subgroup, in which we found an attenuated increase of frailty over time (see Figure 1). This attenuated increase over time differs from population‐based studies showing linear time effects (as we also found in the non‐depressed group) or exponential increases in frailty in the latest stages of life.^26^ Several explanations can be put forward to explain this discrepancy. First, selective dropouts, as more patients in the depressed subgroup died during follow‐up compared to the non‐depressed comparison group.^16^ Second, the FI may be falsely inflated at baseline due to confounding by a high depressive symptom severity at baseline. Since depressive symptom severity declines during follow‐up, this bias lessens during follow‐up and could have attenuated the impact of depressive disorder on biological ageing (as discussed below). Lastly, the non‐linear trend that we found is in line with the reliability theory of ageing.^27^ This theory explains why the slope of the FI in relation to age gradually decreases with increasing frailty levels.^28^

### 4.4 Reciprocal associations between depression and frailty

To our knowledge, only three studies have shown that frailty in depression is associated with a protracted course of depressive symptoms over time.^29^, ^30^ All studies assessed frailty according to the Fried Frailty Phenotype. Recently, we showed that within the NESDO sample, the Fried Frailty Phenotype also predicted non‐remission of late‐life depressive disorder according to DSM ‐criteria.^31^ In this latter study, however, we did not adjust for baseline depressive symptom severity. The present study is the first to explore the impact of the FI on the course of late‐life depression. We did not find an association between baseline frailty status and the course of depressive symptoms over time. However, frail‐depressed patients had a lower likelihood of remission during follow‐up, but this was fully explained by a higher severity level of the depressive disorder. Whether or not the presence of frailty has (falsely) inflated self‐reported depressive symptom severity, thus partially explaining this negative finding due to residual confounding, needs further study.

In line with the theory of depression as a condition of accelerated ageing, we found that frailty worsens significantly faster over time in older patients with a depressive disorder compared to their non‐depressed counterparts. Although the underlying mechanisms connecting depression to accelerated ageing are not well understood, depression has been associated with ageing at the molecular level, for example, DNA damage and mitochondrial dysfunction, the cellular level, for example, telomere attrition, protein accumulation and abnormal secretory patterns, and lastly, at the tissue and system level, for example, sarcopenia and metabolic diseases.^32^, ^33^, ^34^, ^35^, ^36^ Our study also connects depression to a clinical level of senescence, namely frailty. From a clinical perspective, depressed older patients should be considered at increased risk of becoming frail and thus constitute a sample suitable for selective prevention of frailty. Since physical activity is of benefit for mood and recurrent depression^37^ as well as frailty^38^ complementing pharmacotherapy or psychotherapy with physical activity seems promising for the treatment of frail‐depressed patients.

### 4.5 Methodological considerations

One of the strengths of our paper is the assessment of depressive disorder according to a structured psychiatric interview combined with a self‐reported depression rating scale that is generally used in population‐based studies. A further strength is the assessment of frailty with the FI. The FI is a method that can be retrospectively operationalised in any dataset that contains sufficient information on health deficits related to several physiological domains.^39^, ^40^ The FI is one of the most dominant and well‐validated frailty models, and the possibility to operationalise an FI retrospectively is generally considered a strength that does not affect the validity of the study. Moreover, since we did not lack any important domain of health deficits, we were even able to exclude health deficits associated with depression to avoid overlap in the constructs of frailty and depression. Nonetheless, we still found some evidence for diagnostic overlap between frailty and depressive symptom severity. First, the predictive value of frailty for remitted depression at follow‐up was fully explained by the severity of the depressive disorder at baseline. Second, the cross‐sectional association between the NESDO‐FI and chronological age was the lowest at baseline assessment within the depressed subgroup (see Appendix SA). This may indicate confounding, since depressive symptom severity was the highest at baseline and depressive symptom severity, unlike frailty severity, is not associated with age.^41^ Lastly, the increase of frailty over time decelerated around the 4‐year follow‐up, whereas in population‐based cohort studies, a linear or even exponential increase has been reported (as discussed above). These findings fit with previous studies in population‐based cohorts in which high correlations have been found between frailty and self‐report depressive symptoms.^9^ Our results, therefore, argue for the use of DSM or ICD ‐criteria to define depressive disorder when studying its relationship with frailty.

Some methodological issues need to be addressed. First, of the eligible patients, 48.7% gave informed consent to participate in the NESDO study.^15^ Although frailty indicators are not available of non‐respondents, it is likely that these patients were more frail, and the difference in frailty between depressed patients and their non‐depressed counterparts may have been underestimated in the present study. Second, the FI is partly based on subjective health measures like self‐report disabilities assessed with the WHO‐Disability Assessment Schedule (WHO‐DAS, see Appendix SA). These health deficits may be confounded by depression due to cognitive biases of depressed patients when evaluating subjective health states.^42^ This could explain why the FI is partly confounded by depressive symptom severity, even though mood‐related items were excluded. Third, since frailty is associated with mortality,^20^ prospective studies on the course of frailty will be biased due to a healthy survivor effect. Although linear mixed models account for missing data, the ageing effect of depressive disorder may have been underestimated.

### 4.6 Final conclusion

Our results contribute to a better understanding of the reciprocal relationship between frailty and depression in later life. Since frailty is highly prevalent among depressed older patients, depressive disorder accelerates the development of frailty over time, and frailty in depression interferes with mental and somatic health outcomes, it is relevant to better address frailty in both treatment studies on late‐life depression^43^ and clinical care. In other areas of medicine, multidisciplinary geriatric care models have proven benefit for older patients with chronic conditions regarding adverse health effects and quality of life.^44^

## CONFLICT OF INTEREST

The authors have no personal or financial conflicts of interest.

## ETHICS APPROVAL STATEMENT

The authors assert that all procedures contributing to this work comply with the ethical standards of the relevant national and institutional committees on human experimentation and with the Helsinki Declaration of 1975, as revised in 2008. The protocol of the NESDO study was approved centrally by the Ethical Review Board of the VU University Medical Center.

## AUTHOR CONTRIBUTIONS

All authors satisfy the four conditions of the ICMJE for an authorship by having substantially contributed to the concept of the study, interpretation of the data, critical revision of the article, approval of the final version and agreed to be accountable for all aspects of the work. Richard C. Oude Voshaar, Menelaos Dimitriadis and Rob H.S. van den Brink have analysed the data. Richard C. Oude Voshaar and Menelaos Dimitriadis have drafted the first version. Didi Rhebergen and Hans W. Jeuring supervised the research.

## Supporting information

Supplementary Material

## Data Availability

Data of the NESDO study are available for other research groups depending on an a priori defined research question and analysis plan.
