# Supplementary material for: A 6‐year prospective clinical cohort study on the bidirectional association between frailty and depressive disorder
Source: Int J Geriatr Psychiatry. 2021 Jun 19;36(11):1699–707. doi: 10.1002/gps.5588 (PMC8596411; doi:10.1002/gps.5588)
Supplement: Supplementary file 1 — Supplementary Material [file GPS-36-1699-s001.docx]

**Appendix 1**

Health deficits and their coding included in the NESDO Frailty Index (FI)

| **Item** | **Potentially relevant health deficit** | **Cut-off values (including scoring rules)#** |
| --- | --- | --- |
| *Chronic diseases (14 items)* | |  |
| 1 | Heart disease | No=0, yes=1 |
| 2 | Peripheral artery disease | No=0, yes=1 |
| 3 | Stroke | No=0, yes=1 |
| 4 | Diabetes | No=0, yes=1 |
| 5 | Pulmonary disease | No=0, yes=1 |
| 6 | Rheumatism (incl. osteoarthritis) | No=0, yes=1 |
| 7 | Cancer | No=0, yes=1 |
| 8 | Ulcer | No=0, yes=1 |
| 9 | Intestinal disease | No=0, yes=1 |
| 10* | Liver disease | No=0, yes=1 |
| 11 | Epilepsy | No=0, yes=1 |
| 12 | Thyroid disease | No=0, yes=1 |
| 13 | Hypertension | No=0, yes=1 |
| 14 | Polypharmacy (Nmed) | 0 – 2 prescribed drugs = 0  3 or 4 drugs = 0.5  ≥5 drugs = 1 |
| *Physical performance (objective)* | |  |
| 15 | Body mass index | 18.5 – 25 = 0  >25 – 30 = 0.5  <18.5 or >30 = 1 |
| 16 | Waist circumference (WC) | WC <88 cm (men) and <102 (women) = 0  WC ≥88 cm (men) or ≥102 cm (women) = 1 |
| 17 | Systolic Blood Pressure (SBP) | SBP 90-140 = 0  SBP 140-160 = 0.5  SBP, < 90 = 0.5  SBP > 160 = 1 |
| 18 | Gait speed (s) | Male, ≤173 cm or female ≤159 cm: <9s = 0  ≥9s = 1  Male, >173 cm or female ≥159 cm: <8s = 0  ≥8s = 1  Not able to walk = 1 |
| 19 | Handgrip strength (kg) | Male, BMI ≤24 ≤29 kg = 1  BMI >24 ≤26 ≤30 kg = 1  BMI >26 ≤28 ≤30 kg = 1  BMI >28 ≤32 kg = 1  Female, BMI ≤23 ≤ 17 kg = 1  BMI >23 ≤26 ≤ 17.3 kg = 1  BMI >26 ≤29 ≤ 18 kg = 1  BMI >29 ≤ 21 kg = 1 |
| *Physical and cognitive performance (subjective, all items of the WHO-DAS)* | | |
| 20 | Concentrating or doing something for 10 minutes | None = 0  Mild = 0.25  Moderate = 0.50  Severe = 0.75  Extreme / cannot do = 1 |
| 21 | Standing for long periods such as 30 minutes | None = 0  Mild = 0.25  Moderate = 0.50  Severe = 0.75  Extreme / cannot do = 1 |
| 22 | Walking a long distance such as a kilometre | None = 0  Mild = 0.25  Moderate = 0.50  Severe = 0.75  Extreme / cannot do = 1 |
| 23 | Standing up from sitting down | None = 0  Mild = 0.25  Moderate = 0.50  Severe = 0.75  Extreme / cannot do = 1 |
| 24 | Washing your whole body | None = 0  Mild = 0.25  Moderate = 0.50  Severe = 0.75  Extreme / cannot do = 1 |
| 25 | Getting dressed | None = 0  Mild = 0.25  Moderate = 0.50  Severe = 0.75  Extreme / cannot do = 1 |
| 26 | Eating | None = 0  Mild = 0.25  Moderate = 0.50  Severe = 0.75  Extreme / cannot do = 1 |
| *Blood biomarkers* | |  |
| 27 | Kreatinine | Male: 50-110 umol/L = 0, Other values = 1  Female: 50-90 umol/L = 0, Other values = 1 |
| 28 | Hb | Male: < 8.5 = 1  Female < 7.5=1 |
| 29 | Albumin | ≥ 40 g/L = 0  < 40 g/L = 1 |
| 30 | HDL cholesterol | HDL ≥ 1.55 = 0  HDL < 1.55 = 1 |
| 31 | LDL cholesterol | LDL < 2.59 = 0  LDL 2.59 – 3.34 = 0.5  LDL > 3.34 = 1 |
| 32* | Triglycerides | TG <2.0 mmol/L = 0  TG ≥2.0 mmol/L = 1 |
| 33 | Fasting glucose | < 6.1 = 0  ≥ 6.1 = 1 |
| 34 | TSH | TSH 0.5 – 4.0 mU/L = 0  TSH <0.5 mU/L = 1  TSH <4.0 mU/L = 1 |
| *Sensory functioning (2 items)* | |  |
| 35 | Vision (do you see well enough?) | Yes, without difficulty = 0  Yes, with some difficulty = 0.33  Yes, with much difficulty = 0.67  No, I cannot = 1 |
| 36 | Hearing (do you hear well enough?) | Yes, without difficulty = 0  Yes, with some difficulty = 0.33  Yes, with much difficulty = 0.67  No, I cannot = 1 |
| *Mental functioning (1 item)* | |  |
| - | Depressed mood (IDS item 5) | NOT INCLUDED (TO AVOID OVERLAP WITH DEPRESSION) |
| Subjective health measures (5 items) | |  |
| 37 | Pain (Chronic Pain Grade) | Pain free (0) = 0  Grade I = 0.25  Grade II = 0.50  Grade III = 0.75  Grade IV = 1 |
| 38* | Feeling anxious or tense (IDS item 7) | I do not feel anxious or tense (1) = 0  I feel anxious or tense less than half of the time (2) = 0.33  I feel anxious or tense more than half of the time (3) = 0.67  I feel extremely anxious or tense nearly all of the time (4) = 1 |
| 39* | Falling asleep (IDS item 1) | I never take >30 min to fall asleep (1) = 0  I take at least 30 min to fall asleep, less than half of the time (2) = 0  I take at least 30 min to fall asleep, > half of the time (3) = 0.5  I take more than 60 min to fall asleep, < half of the time (4) =1 |
| 40* | Sleep during the night (IDS item 2) | I do not wake up at night (1) = 0  I have a restless, light sleep with a few brief awakeing each night (2) = 0  I wake up at least once a night, but I go back to sleep easily (3) = 0  I awaken more than once a night and stay awake for >20 min, more than half of the time (4) = 1 |
| 41* | Do you have motivation (AS item 7) | A lot (0) = 0  Some (1) = 0.33  Slightly (2) = 0.67  Not at all (3) = 1 |
| 42* | Do you have energy for daily activities (AS item 8) | A lot (0) = 0  Some (1) = 0.33  Slightly (2) = 0.67  Not at all (3) = 1 |
| *Cognitive functioning (6 items)* | |  |
| 43 | Sum score MMSE | Unimpaired >25 = 0  Impaired ≤ 25 =1 |
| 44 | STROOP processing speed | Scoring worse than lowest decile of baseline control group = 1 |
| 45 | STROOP interference control | Scoring worse than lowest decile of baseline control group = 1 |
| 46 | Working memory (digit span) | Scoring worse than lowest decile of baseline control group = 1 |
| 47 | 10-WT - Immediate recall | Scoring worse than lowest decile of baseline control group = 1 |
| 48 | 10-WT - Delayed recall | Scoring worse than lowest decile of baseline control group = 1 |

Abbreviations: WHO-DAS, World Health Organisation Disability Scale; AS, Apathy Scale; MMSE, Mini Mental State Examination; 10-WT, 10-word test for verbal memory; IDS, Inventory of Depressive Symptoms;

* Excluded in the final frailty index for the following reasons. First, liver disease (item 10) was excluded because of a

prevalence of less than 1% at baseline. Second, we excluded six variables that had a negative cross-sectional association with age and improved over time. These variables were triglycerides (item 32), feeling anxious or tense (item 38), sleep onset (item 39), sleep quality (item 40), motivation (item 41), and level of energy (item 42). This make sense since the latter five items are intrinsically related to depression.

# When clinically relevant, deficits should be coded as any number between 0 and 1 (e.g. 0 - 0.25 – 0.50 – 0.75 – 1),

because progressive insight has shown that this yields a more sensitive frailty measure.
